# Supplementary material for: Complexome profiling of the Chlamydomonas psb28 mutant reveals TEF5 as an early PSII assembly factor
Source: Plant Cell. 2025 Mar 17;37(6):koaf055. doi: 10.1093/plcell/koaf055 (PMC12164590; doi:10.1093/plcell/koaf055)
Supplement: koaf055_Supplementary_Data [file koaf055_supplementary_data.zip › psb28_interactive.zip!/plots/intensity/qualityPlots/peptides/_Cre01.g045100.t1.2.html]

### Cre01.g045100.t1.2

Trivial name:   
SeqCoverage [median]: 23.30  
Mass: 20.90 kDa  
  
 

| Sequence | MissedCleavage | Mass [Da] | wtIntensity | muIntensity | Score |
| --- | --- | --- | --- | --- | --- |
| FLGDEETEKNENAGVAAR | 1 | 1948.91 | 0.00 | 624.91 | 43.30 |
| MAVLSDTLQYEPDHP | 0 | 1714.79 | 759.70 | 2753.19 | 142.08 |
| VAAYESPLDFR | 0 | 1266.62 | 0.00 | 1554.14 | 92.06 |
|  |  |  | 759.7 | 4932.2 |  |
